# Aircraft Detection from VHR Images Based on Circle-Frequency Filter and Multilevel Features

**DOI:** 10.1155/2013/917928

**Published:** 2013-09-15

**Authors:** Feng Gao, Qizhi Xu, Bo Li

**Affiliations:** ^1^Beijing Key Laboratory of Digital Media, School of Computer Science and Engineering, Beihang University, Beijing 100191, China; ^2^State Key Laboratory of Virtual Reality Technology and Systems, Beihang University, Beijing 100191, China

## Abstract

Aircraft automatic detection from very high-resolution (VHR) images plays an important role in a wide variety of applications. This paper proposes a novel detector for aircraft detection from very high-resolution (VHR) remote sensing images. To accurately distinguish aircrafts from background, a circle-frequency filter (CF-filter) is used to extract the candidate locations of aircrafts from a large size image. A multi-level feature model is then employed to represent both local appearance and spatial layout of aircrafts by means of Robust Hue Descriptor and Histogram of Oriented Gradients. The experimental results demonstrate the superior performance of the proposed method.

## 1. Introduction

With the advent of the very high-resolution (VHR) earth observation satellite programs, the spatial resolution of remote sensing images dramatically increased from tens of meters to tens of decimeters, such as QuickBird, GeoEye-1, and WorldView-2. High-resolution images of a small number of locations are publicly available via Google Earth at an astonishing ground sampling distance (GSD) of 0.15 meter [1]. The high-resolution satellite sensors can acquire high definition images of ground objects with abundant spatial details and contextual information, which make it feasible to detect and recognize artificial targets, such as aircraft [2, 3], vehicle [1, 4, 5], ship [6], and building [7, 8]. In this paper, we concentrate on the problem of detecting aircrafts from such high-resolution aerial and satellite imagery. Aircraft automatic detection from VHR images plays an important role in a wide variety of applications. Detecting and tracking aircrafts in aerial videos is an important component in visual surveillance systems. Images of military airport, along with the distribution of aircrafts, can provide valuable information for military monitoring. However, while image resolution upgrades to decimeter level, background of ground targets becomes complex and disturbing. It is therefore a challenging task to detect ground targets in a cluttered background. The presence of complex structures, such as buildings and airport terminals, can cause many false alarms.

Various methods have been developed for object detection from remote sensing images. For example, Lei et al. [9] proposed a color-enhanced rotation-invariant Hough forest method for detecting aircrafts and buildings. Liu et al. [2] presented a coarse-to-fine method by integrating shape prior; the pose of an aircraft is roughly estimated by template matching, and a shape prior is used to segment the target. Li et al. [10] proposed detecting aircrafts using a contour-based spatial model. Inglada [11] proposed a supervised learning method which used geometric image features to characterize classes of objects. Akçay and Aksoy [12] presented a geospatial object detection method by using hierarchical segmentation which combined spectral and structural information. Recently, there are also some methods concentrating on detecting objects in synthetic aperture radar (SAR) image [13, 14] and hyperspectral image [15, 16].

Another group of methods utilizs a sliding window approach. In this approach, a classifier is first trained to recognize an object; a detection window is scanned over the image; then the classifier is used to determine whether the detection window contains an object or not. Kembhavi et al. [1] proposed a vehicle detector by using partial least square analysis to project high-dimensional features to a much lower dimensional subspace, and then the computational burden can be reduced. Sun et al. [17] presented an aircraft detector which applied a mapping strategy to encode the geometric information in the detection window. Zhang et al. [18] proposed a method to detect aircrafts by encoding the features of the rotated parts and objects. These methods rely on intensity-based features to capture the appearance of the object and obtain good results. However, all the pixels contained in the image are generally searched in these methods, thus resulting in a number of false positive detections. Moreover, due to the variability of color in a cluttered background, color cues have been ignored by most of these detection methods.

**Figure 1:**
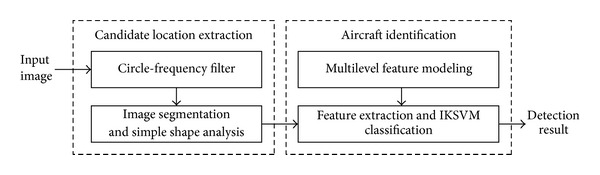
Flow diagram of the proposed method.

The proposed method improves the existing methods by exploiting circle-frequency filter (CF-filter) [3] to extract potential location of aircraft targets from the entire image. Consequently, most of the cluttered background regions are eliminated, and false alarms are therefore greatly reduced. Moreover, a multilevel feature model that incorporates both color and shape information is designed to identify aircrafts by checking potential locations. Our method includes a coarse-to-fine search process. The flow diagram of our method is shown in Figure 1.

**Stage 1 (candidate location extraction by circle-frequency filter)**

Inspired by [3], CF-filter is applied to extract the candidate locations of aircraft. The goal of candidate extraction is to reduce the false positives, for example, man-made objects that may be detected as aircrafts, and false negatives.

**Stage 2 (aircraft identification by multilevel features)**

Robust Hue Descriptor [19] and Histogram of Oriented Gradients [20] are used to capture color and shape information of aircrafts, respectively. Meanwhile, a multilevel feature model is constructed to represent both local appearance and spatial layout of aircrafts.

We implement the proposed method on remote sensing images collected from aerial and WorldView-2 satellite images. Our methods are also compared with two previously proposed object detection methods, and experimental results demonstrate the superior performance of the proposed method.

## 2. Extraction of Aircraft Candidate Locations

The candidate location extraction is comprised of two steps: circle-frequency filter (CF-filter) and candidate locations extraction. To facilitate discussion, we begin this section with a general introduction to CF-filter. We follow that with an introduction on how candidate locations can be extracted. 

### 2.1. Circle-Frequency Filter

**Figure 2:**
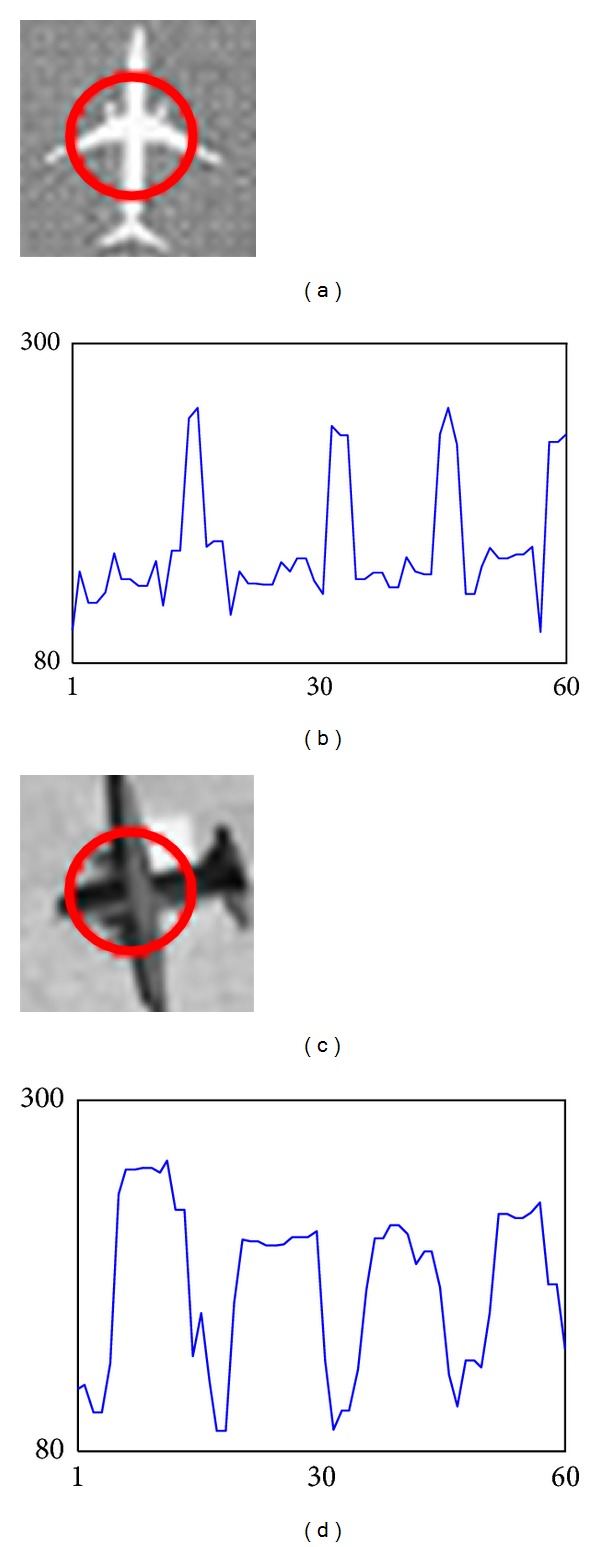
Illustration of the aircraft regular patterns. (a) Bright aircraft (32 × 32 pixels); (b) regular pattern of the bright aircraft; (c) dark aircraft (32 × 32 pixels); (d) regular pattern of the dark aircraft. The intensities along the red circle will change regularly from darkness to brightness and will repeat four times.

The candidate extraction is based on two common features of aircraft as follows. First, airplanes and their background have difference in brightness. Second, airplanes are comprised by four main bulges [3], that is, head, tail, and two wings. If a circle is located at the center of an aircraft, the intensities along the circle will change regularly from darkness to brightness and will repeat four times. Figures 2(a) and 2(c) are two examples of typical airplanes, that is, civil and military aircraft. In general, civil aircraft is brighter than background, while military aircraft is darker than background. Figures 2(b) and 2(d) illustrate the regular patterns of the two aircrafts.

Assume that *f*_*i*_   (*i* = 0,…, *N* − 1) are pixel values along the circle centered at (*x*, *y*) with radius *r*, the CF-filter can be expressed as

(1) $$\begin{array}{c} f\left(x,y\right)={\left(\sum_{k=0}^{N-1}f_{k}\cos\left(8\pi \frac{k}{N}\right)\right)}^{2}+{\left(\sum_{k=0}^{N-1}f_{k}\sin\left(8\pi \frac{k}{N}\right)\right)}^{2}. \end{array}$$

**Figure 3:**
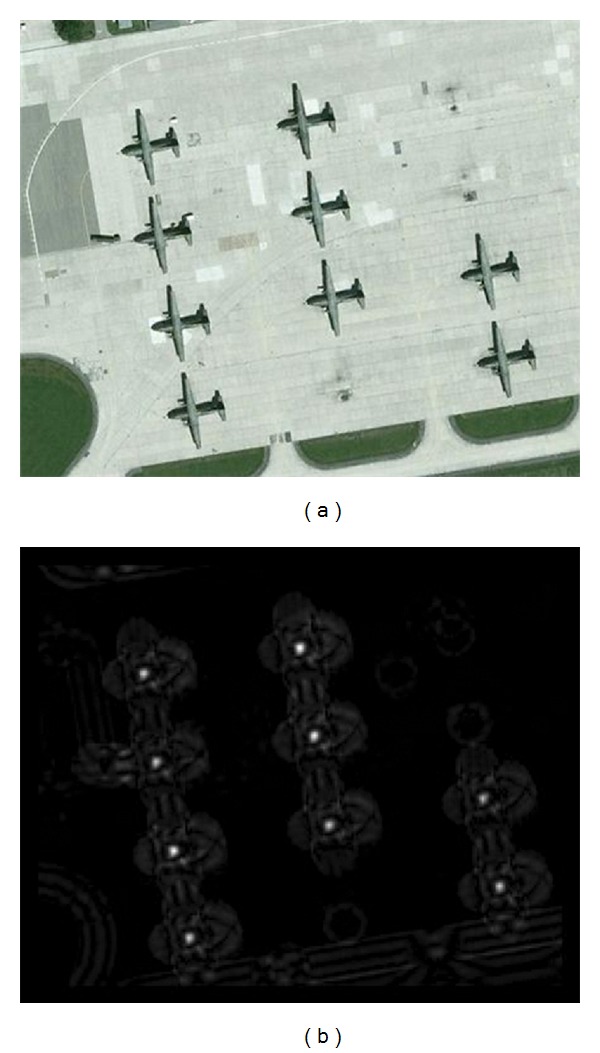
Illustration of CF-filter. (a) Original image; (b) CF-filtered result.

If the whole image is filtered by CF-filter, a bright spot will be generated at the center of an airplane.  Figure 3 gives an example of a CF-filtered image, where the values of CF-filtered image are normalized to (0, 255). It can be seen from Figure 3 that only the center of airplane has a strong response. The CF-filter is rotation invariant; thus, airplanes with different layouts can be detected by CF-filter.

### 2.2. Aircraft Candidate Locations Extraction

As mentioned before, only the center of aircraft has a strong response in the CF-filtered image. Therefore, aircraft candidates can be extracted by removing low values of CF-filtered image. Here, Otsu's method [21] is used to calculate a global adaptive threshold *T* from CF-filtered image to remove low values, most of which are produced by clutters within background.

According to the specific size of aircraft candidate (bright spot), a simple shape analysis process is applied to eliminate obvious false candidates. The pixels remained are considered as true candidates; then these regions are forwarded to the second stage.

## 3. Identifying Aircrafts by Multilevel Features

The appearance of an aircraft can be better captured when color and shape information is combined together. In the second stage, two classes of feature are involved in the proposed solution: Robust Hue Descriptor and Histogram of Oriented Gradients. We build a multilevel feature model, which is akin to the image pyramid representation proposed by Lazebnik et al. [22], to represent both local appearance and spatial layout of an aircraft.

### 3.1. Robust Hue Descriptor

Color is an indispensable aspect in describing the world around us. However, in a cluttered background, the color representation of an object is greatly interfered by photometric variance; thus, most of existing methods avoid exploiting color information. To obtain a good color descriptor, Van De Weijer et al. [19] proposed robust hue (HUE) descriptor. Experiments show that this descriptor is reliable under photometric and geometrical changes in image retrieval and classification. The HUE descriptor is represented by a histogram over hue computed from the corresponding RGB values of each pixel according to

(2) $$\begin{array}{c} \text{hue}=\text{arc tan}\left(\frac{\sqrt{3}\left(R-G\right)}{R+G-2B}\right). \end{array}$$

The HUE descriptor is invariant with respect to lighting geometry. In our implementation, the HUE descriptor has 6 dimensions.

### 3.2. Histogram of Oriented Gradients

Histograms of Oriented Gradients (HOG) [20] are used to capture the spatial distribution of gradients. Since the HOG operator has been proven to be robust and reliable for representing the shape of an object, it is therefore involved into our feature model to describe the shape information of aircrafts. In Dalal's work, the detection window of the HOG operator is split into small cells, in which a 9-bin histogram of gradient orientations is calculated. Every 2 × 2 cells are grouped together to form a block; thus, each block has a 36-bin histogram feature. Four blocks of features are then concatenated to form the HOG descriptor. Maji et al. [23] proposed a Spatial Histogram of Oriented Gradients (SPHOG) descriptor. Compared to Dalal's HOG descriptor, Maji's descriptor computes the oriented edge energy response by the magnitude of odd elongated oriented filters. Experiments show that, while combining with intersection kernel SVM (IKSVM), the SPHOG descriptor outperforms the original single scale HOG. Hence, we follow Maji's method and use the odd elongated oriented filters to compute oriented edge energy and SPHOG descriptor.

### 3.3. Multilevel Feature Model

We build a multilevel feature model to represent the color and shape information of aircrafts. The detection window *W*_*i*_ is decomposed into a sequence of increasingly finer spatial cells by repeatedly doubling the number of decompositions. For cell *C*_*i*_, the feature representation is obtained by concatenation:

(3) $$\begin{array}{c} C_{i}=\left[{\text{HUE}}_{i},{\text{HOG}}_{i}\right]. \end{array}$$

**Figure 4:**
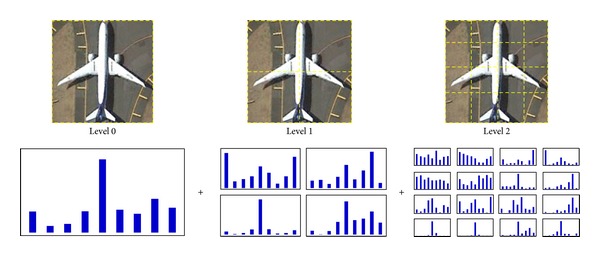
Multilevel feature representation.

The feature vector for each cell is a 15-dimensional vector (HUE descriptor has 6 dimensions, and HOG descriptor has 9 dimensions). The number of pixels assigned to a specified color bin or gradient bin is the sum over those contained in the four cells it is divided into at a finer level. Hence, the multilevel feature model is a pyramid representation. The final features are the concatenations of all the 15-dimensional feature vectors. As shown in Figure 4, the cell at level *l* has 2^*l*^ cells along each dimension. Therefore, level 0 is represented by a 15-dimensional vector, level 1 by a 60-dimensional vector, and so forth. Total features of the detection window are a vector with dimensionality 15 × ∑_*l*∈*L*_4^*l*^. Through a multilevel representation, the feature model captures both the local image appearance and its spatial layout. In our implementation, we use a feature model with three levels, yielding a total of 21 cells.

In this paper, SVM with histogram intersection kernel (IKSVM) [23] is applied as a classifier. For feature vectors **x**, **y** ∈ ℝ_+_^*n*^, the intersection kernel *k*(**x**, **y**) can be expressed as

(4) $$\begin{array}{c} k\left(x,y\right)=\sum_{i=1}^{n}\;\min\left(x\left(i\right),y\left(i\right)\right), \end{array}$$

and the classification is based on evaluating

(5) $$\begin{array}{c} h\left(x\right)=\sum_{l=1}^{m}\alpha_{l}y_{l}k\left(x,x_{l}\right)+b. \end{array}$$

Maji et al. [23] noticed that the summation in (5) can be reformed as

(6) h(x)=∑i=1n αlyl(∑l=1m min⁡(x(i),xl(i)))+b=∑i=1n(∑l=1m αlylmin⁡(x(i),xl(i)))+b=∑i=1n  hi(x(i))+b.

Consider the functions *h*_*i*_(*s*) at fixed point *i*; ${\overset{-}{x}}_{l}(i)$ represents the sorted values of *x*_*l*_(*i*) in increasing order with corresponding values of *α* and labels given by ${\overset{-}{\alpha }}_{l}$ and ${\overset{-}{y}}_{l}$. Let *r* be the largest integer, such that ${\overset{-}{x}}_{r}(i)\leq s$; then we can get

(7) hi(s)=∑l=1m αlylmin⁡(s,xl,i)

(8) =∑1≤l≤rα−ly−lx−l(i)+s∑r<l≤mα−ly−l

(9) =Ai(r)+sBi(r),

where $A_{i}(r)=\sum_{1\leq l\leq r}{\overset{-}{\alpha }}_{l}{\overset{-}{y}}_{l}{\overset{-}{x}}_{l}$, $B_{i}(r)=\sum_{r<l\leq m}{\overset{-}{\alpha }}_{l}{\overset{-}{y}}_{l}$. It is obvious that (8) is piecewise linear and the function *A*_*i*_ and *B*_*i*_ are independent of the input data. Therefore, *s* can be precomputed by finding the position of *s* = *x*(*i*) in the sorted list. Maji et al. [23] noticed that the support distributions in each dimension tend to be smooth and concentrated. Therefore, *h*(*x*) can be approximated by simpler functions, and the prediction can be accelerated. In this paper, *h*_*i*_(*s*) is computed by a look-up table with a piecewise constant approximation.

## 4. Experimental Results and Evaluation

### 4.1. Data Collection and Training

**Figure 5:**
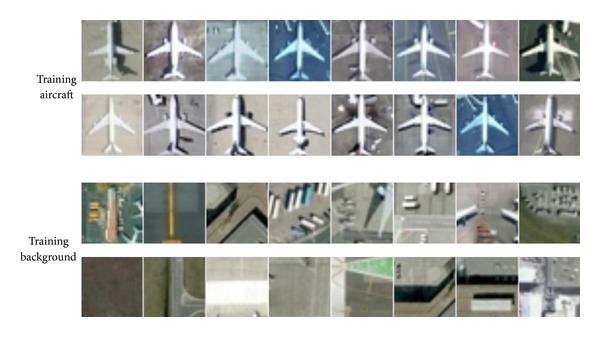
Examples of positive and negative image patches of training dataset.

From the publicly available Google Earth service and WorldView-2 satellite images, we collected 914 positive image patches (aircrafts) and 1953 negative image patches. Negative image patches are randomly chosen from the airport backgrounds. Each image patch has a size of 32 × 32 pixels.  Figure 5 shows parts of training image patches of the dataset. We build our testing set by collecting 214 images of other airports containing 597 aircrafts in China, Germany, and France. Each image contains multiple instances of aircrafts with different orientations and sizes.

**Figure 6:**
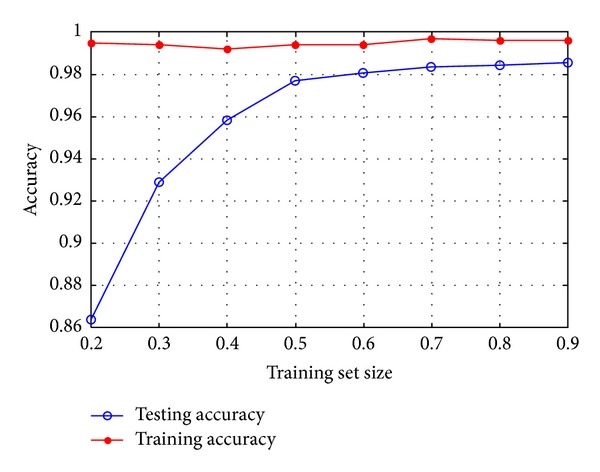
Cross-validation results.

The IKSVM [23] is applied as a classifier in our method. To find an optimal training set size, we carried out cross-validation. A portion of the training set is selected to train the IKSVM classifier, and then the performance of the classifier is tested by the remaining images in the training set. This process is repeated, and average accuracy is calculated. The average training accuracy and testing accuracy versus training set size are illustrated in Figure 6. The training accuracy is high for all training set sizes, indicating that the multilevel feature model is effective to distinguish the images in the training set. The testing accuracy tends to be stable at a high rate when the number of training images is more than half of training set, which indicates that our method is capable of representing aircraft from a small training set.

### 4.2. Quantitative Evaluation

We manually label the aircrafts appearing in all testing images as a ground truth. When a detection to be marked is a true positive, more than 50% of it must be detected. *M* denotes the total number of aircrafts in testing images. The recall-precision curve (RPC) is chosen to exhibit the tradeoff between recall and precision. Recall and 1 − precision are defined as

(10) $$\begin{array}{c} \text{Recall}=\frac{\text{TP}}{\left(\text{TP}+\text{FN}\right)},\\ 1-\text{Precision}=\frac{\text{FP}}{\left(\text{TP}+\text{FP}\right)}, \end{array}$$

where TP (true positive) denotes the number of true detected aircrafts, FN (false negative) is the number of missed detections, and FP (false positive) is the number of false detected aircrafts. Therefore, recall denotes the number of true detected aircrafts divided by the total number of aircrafts in testing images and 1 − precision denotes the number of false detected aircrafts divided by the elements detected. The ratio recall/(1 − precision) is used to represent the performance of the algorithm.

Since the size of aircraft in the testing set is unknown, the testing images are scanned at multiple scales. At each scale, the radius *r* and the number of pixels *N* in CF-filter are set to 8 and 60, respectively. After candidate extraction, we use the sliding window approach to detect aircrafts. The size of the sliding window is 32 × 32 pixels, and the step is set to be 8 pixels.

**Figure 7:**
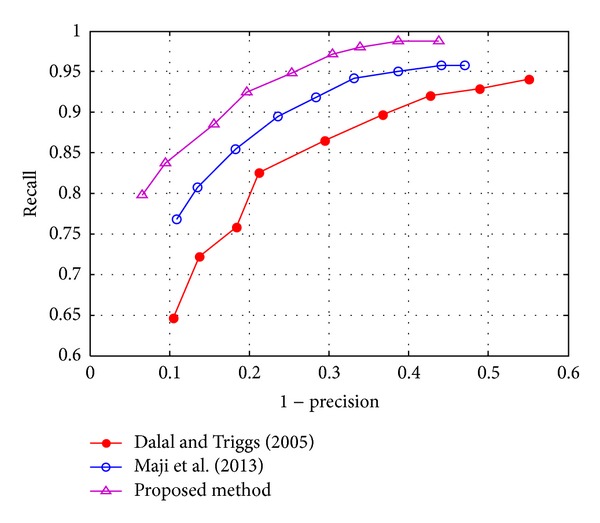
Performance of the three detectors.

We compared our method with two classical methods. The first method is proposed by Dalal and Triggs [20], in which a linear Support Vector Machine is exploited to classify aircrafts and background by HOG features. The second method is Maji's approach [23], where the IKSVM is applied as a classifier and spatial histograms of oriented gradients (SPHOG) are calculated for the classifier. From Figure 7, we can observe that the proposed method outperforms the other two detectors. At the same recall level, the precision of the proposed method is the best, which means that the false alarms rate of the proposed method is the lowest. While at the same precision level, the proposed method detects more true positives.

**Figure 8:**
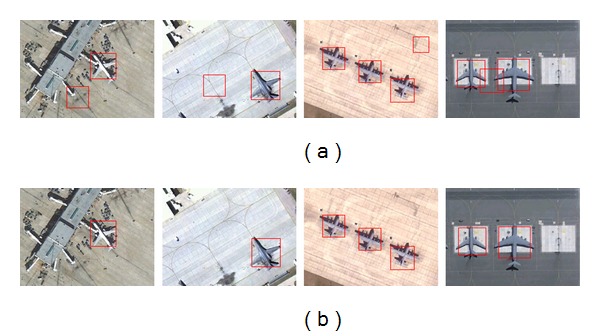
The effectiveness of CF-filter. (a) Detection results without CF-filter; (b) detection results with CF-filter.

In order to test the impact of CF-filter, our original method is compared to the modified method without CF-filter, see Figure 8. The first row is the experimental results without CF-filter and the second row is the results with CF-filter. It is obvious that false positives of our original method are much less than the modified method. This is because CF-filter can identify the shape of aircrafts in cluttered background.

## 5. Conclusion and Future Work

In this paper, we proposed a coarse-to-fine method for aircraft detection in VHR remote sensing images. CF-filter is applied to extract the candidate locations of aircrafts in the coarse stage. In the fine stage, a multilevel feature model is designed to capture both the local appearance and spatial layout of aircrafts. Meanwhile, Robust Hue Descriptor and Histogram of Oriented Gradients are utilized to describe the color and shape information of aircrafts. The experiment results show the good performance of the proposed method.

Many satellite images provide more than red, green, and blue channels; other channels such as infrared, coastal blue, and red edge are often acquired. In the future work, we will study how to utilize more spectral information to represent aircrafts.

## References

[1] Kembhavi A, Harwood D, Davis LS (2011). Vehicle detection using partial least squares. *IEEE Transactions on Pattern Analysis and Machine Intelligence*.

[2] Liu G, Sun X, Fu K, Wang H, Aircraft recognition in high-resolution satellite images using coarse-to-fine shape prior (2013). *IEEE Geoscience and Remote Sensing Letters*.

[3] Cai H, Su Y Airplane detection in remote-sensing image with a circle-frequency filter.

[4] Salehi B, Zhang Y, Zhong M (2012). Automatic moving vehicles information extraction from single-pass worldView-2 imagery. *IEEE Journal of Selected Topics in Applied Earth Observations and Remote Sensing*.

[5] Leitloff J, Hinz S, Stilla U (2010). Vehicle detection in very high resolution satellite images of city areas. *IEEE Transactions on Geoscience and Remote Sensing*.

[6] Zhu C, Zhou H, Wang R, Guo J (2010). A novel hierarchical method of ship detection from spaceborne optical image based on shape and texture features. *IEEE Transactions on Geoscience and Remote Sensing*.

[7] Chen L, Zhao S, Han W, Li Y (2012). Building detection in an urban area using lidar data and QuickBird imagery. *International Journal of Remote Sensing*.

[8] Ok AO, Senaras C, Yuksel B (2013). Automated Detection of arbitrarily shaped buildings in complex environments from monocular VHR optical satellite imagery. *IEEE Transactions on Geoscience and Remote Sensing*.

[9] Lei Z, Fang T, Huo H, Li D (2012). Rotation-invariant object detection of remotely sensed images based on texton forest and hough voting. *IEEE Transactions on Geoscience and Remote Sensing*.

[10] Li Y, Sun X, Wang H, Sun H, Li X (2012). Automatic target detection in high-resolution remote sensing images using a contour-based spatial model. *IEEE Geoscience and Remote Sensing Letters*.

[11] Inglada J (2007). Automatic recognition of man-made objects in high resolution optical remote sensing images by SVM classification of geometric image features. *ISPRS Journal of Photogrammetry and Remote Sensing*.

[12] Akçay HG, Aksoy S (2008). Automatic detection of geospatial objects using multiple hierarchical segmentations. *IEEE Transactions on Geoscience and Remote Sensing*.

[13] Atteia GE, Collins MJ (2013). On the use of compact polarimetry SAR for ship detection. *ISPRS Journal of Photogrammetry and Remote Sensing*.

[14] Alonso MT, Lopez-Martinez C, Mallorqui JJ, Salembier P (2011). Edge enhancement algorithm based on the wavelet transform for automatic edge detection in SAR images. *IEEE Transactions on Geoscience and Remote Sensing*.

[15] Renard N, Bourennane S (2008). Improvement of target detection methods by multiway filtering. *IEEE Transactions on Geoscience and Remote Sensing*.

[16] Bourennane S, Fossati C, Cailly A (2011). Improvement of target-detection algorithms based on adaptive three-dimensional filtering. *IEEE Transactions on Geoscience and Remote Sensing*.

[17] Sun H, Sun X, Wang H, Li Y, Li X (2012). Automatic target detection in high-resolution remote sensing images using spatial sparse coding bag-of-words model. *IEEE Geoscience and Remote Sensing Letters*.

[18] Zhang W, Sun X, Fu K, Wang C, Wang H (2013). Object detection in high-resolution remote sensing images using rotation invariant parts based model. *IEEE Geoscience and Remote Sensing Letters*.

[19] Van De Weijer J, Gevers T, Bagdanov AD (2006). Boosting color saliency in image feature detection. *IEEE Transactions on Pattern Analysis and Machine Intelligence*.

[20] Dalal N, Triggs B Histograms of oriented gradients for human detection.

[21] Otsu N (1979). A threshold selection method from gray-level histograms. *IEEE Transactions on Systems, Man and Cybernetics*.

[22] Lazebnik S, Schmid C, Ponce J Beyond bags of features: spatial pyramid matching for recognizing natural scene categories.

[23] Maji S, Berg AC, Malik J (2013). Efficient classification for Additive Kernel SVMs. *IEEE Transactions on Pattern Analysis and Machine Intelligence*.

